# Unjustly forgotten scientist Wacław Mayzel (1847–1916)—co-discoverer of somatic mitosis

**DOI:** 10.1007/s13353-021-00644-1

**Published:** 2021-06-17

**Authors:** Janusz Limon, Ewa Bartnik, Janusz Komender

**Affiliations:** 1 Medical University of Gdansk, Gdansk, Poland; 2 Institute of Genetics and Biotechnology, Faculty of Biology, University of Warsaw, Warsaw, Poland; 3 Medical University of Warsaw, Warsaw, Poland

**Keywords:** Animal mitosis, Somatic cell divisions, Somatic mitosis

## Abstract

Descriptions of somatic cell divisions were made as early as the eighteenth and nineteenth centuries with varying degrees of accuracy. In this paper, we would like to present a forgotten Polish scientist Waclaw Mayzel (1847–1916), who described somatic mitosis in the corneal epithelium of the frog in 1875 almost simultaneously with the recognized discoveries of animal mitosis by Otto Bütschli and plant mitosis by Eduard Strasburger.

Descriptions of cell divisions were made as early as the eighteenth and nineteenth centuries with varying degrees of accuracy. We would like to present a forgotten Polish scientist, Waclaw Mayzel, who described somatic mitosis in the corneal epithelium of the frog almost simultaneously with the recognized discoveries of animal mitosis by Otto Bütschli and plant mitosis by Eduard Strasburger.

Waclaw Mayzel MD, PhD (Fig. 1) was born in Kunów (Ostrowiec County, Poland) on September 12, 1847. Father Joseph and mother nee Minheymer. Mayzel graduated in 1865 from St. Anna grammar school in Cracow and began to study at the School of Medicine of Warsaw University. Still as a student, Mayzel began to work in the Department of Physiology and Histology headed by Henryk Fryderyk Hoyer Senior. In 1870, he received a diploma *cum eximia laude* and got an assistant position in Hoyer’s Department. After 15 years of academic and research work, he was forced by Russian authorities to leave the University. The conditions of Polish scientists in the second half of the nineteenth century were complicated by the Russian invaders. In general, Russian authorities did not favor the development of science by Polish researchers. For these reasons, several talented Polish scientists decided to leave Poland and, for example, Eduard Strasburger (from a Polish family of German origin from the eighteenth century), a colleague of Mayzel from Warsaw University, took a chairman position in the Botanic Chair in the University of Jena and left Warsaw in 1869 (Hryniewiecki 1938; Korohoda 2012). Waclaw Mayzel decided to stay in Warsaw and devoted his time to laboratory diagnostics analyses. He died on April 19, 1916.

**Fig. 1:**
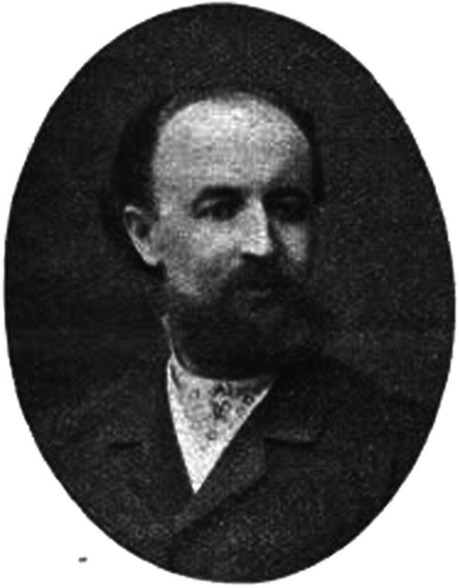
Waclaw Mayzel (1847–1916). Photograph from Gazeta Lekarska nr. 2, p. 44–48, 1907

The main topic of Mayzel’s research was corneal epithelium regeneration in frogs, rabbits, cats, and frog skin. He had observed that nuclei of newly formed cells after division displayed novel structures related to the nucleus. Many researchers at the time believed that cell division involved the “dissolution” of the nucleus with subsequent reassembly of its structure. Bütschli (1875) was the first to identify and order sequentially the stages of nuclear division in several types of animal cells, simultaneously with Mayzel who looked at corneal epithelium mitosis (1875a, 1875b, 1878) and Strasburger’s work (1875; 1876) on the division of plant cells prior to Flemming’s studies on animal cell division who introduced the term *mitosis* (Flemming, 1878). In addition, Bütschli demonstrated that the polar bodies of eggs arise through atypical cell division (Bütschli 1875).

At two meetings of the Warsaw Medical Society held on April 7 and 21, 1874, Mayzel presented the results of his research on epithelium regeneration and its behavior during transplantation. He repeatedly noted numerous coarse grains and filamentous formations in the nuclei of newly formed cells. He concluded that he had found traces of “hitherto unknown details” in his observations and therefore submitted a description of them to the Warsaw Medical Society in a sealed envelope in order to ensure that they would be hidden to ensure his priority in the case of confirmation of these (Mayzel, 1875c; Ostrowska 1975). Shortly afterwards, in 1875, the botanist E. Strasburger [1875] and the zoologist O. Bütschli (1875) published papers in which they linked cell division with the cell nucleus. This “emboldened” Mayzel to send to publish the first descriptions of his observations of mitosis (1875a; 1875b). In March 1876, Mayzel showed his preparations to E. Strasburger, who fully confirmed the great similarity between plant and animal mitoses (Mayzel 1876). In 1884, the memorial book of Prof. H. Hoyer with drawings of animal mitosis made by Mayzel was published (Mayzel 1884) (Fig. 2).

**Fig. 2:**
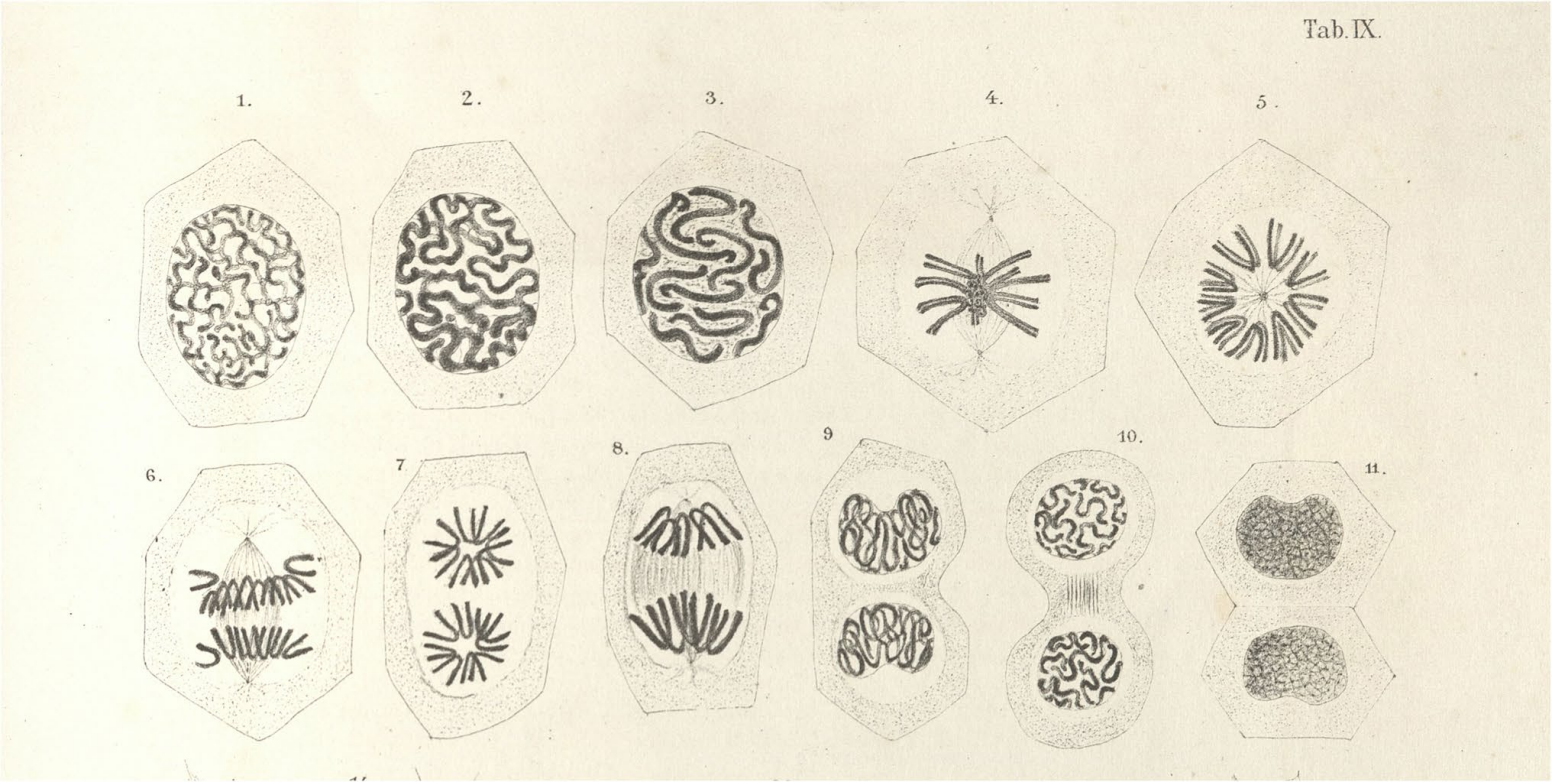
Mitosis of epithelial cells of Urodele larvae. “The figures were drawn on the basis of preparations using the Doyère and Milne-Edwards chamber and then I engraved them in stone” (Mayzel 1884)

Mayzel’s pioneering publication received much attention from many researchers abroad. Many authors confirmed his observations and found several similarities with their findings and widely discussed the presence of several structures in the nucleus and described the process of division of these cells (von Waldeyer, 1876 and 1888; Strasburger 1876; Arnold 1879a; 1879b; Schlecher 1879).

In 1888, W. Waldeyer who introduced the term *chromosomes* stated “I have had the possibility to see the clearest spindles in animal cells recently in the very beautiful preparation of Mayzel” (Waldeyer 1888). Mayzel won the praise of many authors who have quoted portions of the text of his articles and showed his original drawings of mitosis and his methodological protocols. Mayzel extended his research to the tail tissues of triton larvae, salamanders, perch embryos, rabbit, and bird corneas, by observing similar mitotic division figures as in the frog corneal epithelium (Mayzel 1875a; 1875b; 1877). He presented the results of his research at medical congresses in Krakow (1881), Berlin (1881 and 1890), Prague (1882), Paris, London, Rome, and Madrid.

In the history of scientific discovery, the date of publication is critical so it is important to note that the first descriptions of newly discovered karyokinesis were published in 1875 almost simultaneously by Bütschli (May), Strasburger (May), Mayzel (November), and E. v. Beneden (December). Undoubtedly, the input by Mayzel was great and he should be cited like Bütschli (1875) and Strasburger (1876). Later on, Mayzel’s name almost disappeared among the discoverers of mitosis. Much of the blame falls on the Polish histologist W. Szymonowicz, author of the large textbook on histology (1901 first edition) that has had many editions (XII) in five languages. In his textbook in the Polish edition (1924), he lists 15 researchers who deserved the most credit for the explanation of mitosis and does not mention Mayzel’s achievement (Wozniewski 1949; Szumowski 1952). It is hard to believe that the omission of his publication was accidental.

In 1916, Professor A. Sokolowski, secretary of the *Warsaw Medical Society*, wrote: “He left this world… almost completely forgotten, a great Polish scientist, who in different conditions, in a different society, would have been the glory and the ornament of universities and academies…” (Wozniewski 1949).

On the 100th anniversary of the description of mitosis, the Warsaw Medical University issued a bibliophilic print containing the publication of Waclaw Mayzel from 1875b. In 1998, the Medical Faculty of the Polish Academy of Sciences decided to award medical students for their scientific achievements with the “Dr. Waclaw Mayzel Medical Laurel.”

## Data Availability

Not applicable.
