# Correction to: Unjustly forgotten scientist Wacław Mayzel (1847–1916)—co‑discoverer of somatic mitosis

**DOI:** 10.1007/s13353-024-00863-2

**Published:** 2024-04-01

**Authors:** Janusz Limon, Ewa Bartnik, Janusz Komender

**Affiliations:** 1 Medical University of Gdansk, Gdansk, Poland; 2 Institute of Genetics and Biotechnology, Faculty of Biology, University of Warsaw, Warsaw, Poland; 3 Medical University of Warsaw, Warsaw, Poland

**Correction to: Journal of Applied Genetics (2021) 62:639–642**

10.1007/s13353-021-00644-1

The original article contains an error. The article was published with inverted names. Family name was captured first instead of the given names. This should be corrected as follows;

- Janusz Limon^1^, Ewa Bartnik^2^, Janusz Komender^3^

The original article has been corrected.

